# Adult experience of mental health outcomes as a result of intimate partner violence victimisation: a systematic review

**DOI:** 10.3402/ejpt.v5.24794

**Published:** 2014-09-12

**Authors:** Susan Lagdon, Cherie Armour, Maurice Stringer

**Affiliations:** School of Psychology, University of Ulster, Coleraine, Northern Ireland, UK

**Keywords:** Intimate partner violence, domestic violence, PTSD, depression, gender, mental health

## Abstract

**Background:**

Intimate partner violence (IPV) has been known to adversely affect the mental health of victims. Research has tended to focus on the mental health impact of physical violence rather than considering other forms of violence.

**Objective:**

To systematically review the literature in order to identify the impact of all types of IPV victimisation on various mental health outcomes.

**Method:**

A systematic review of 11 electronic databases (2004–2014) was conducted. Fifty eight papers were identified and later described and reviewed in relation to the main objective.

**Results:**

Main findings suggest that IPV can have increasing adverse effects on the mental health of victims in comparison with those who have never experienced IPV or those experiencing other traumatic events. The most significant outcomes were associations between IPV experiences with depression, posttraumatic stress disorder, and anxiety. Findings confirm previous observations that the severity and extent of IPV exposure can increase mental health symptoms. The effect of psychological violence on mental health is more prominent than originally thought. Individual differences such as gender and childhood experience of violence also increase IPV risk and affect mental health outcomes in diverse ways.

**Conclusions:**

Psychological violence should be considered as a more serious form of IPV which can affect the mental health of victims. Experiencing more than one form of IPV can increase severity of outcomes. Researchers should look at IPV as a multi-dimensional experience. A uniformed definition and measure of IPV could help advance knowledge and understanding of this disparaging global issue.

Intimate partner violence (IPV) has become a global issue of concern which negatively affects individuals beyond the immediate harm incurred through physical injury (Giridhar, 2012). Historically, terms such as wife battering, wife abuse, domestic violence, and family violence have been used to describe this occurrence (Van Parys, Verhamme, Temmerman, & Verstraelen, 2014). The narrow focus of some of these previous terms reflects the historical context in which IPV was viewed; with women seen as victims and males as perpetrators. Empirical evidence now demonstrates that both men and women can be both victims and perpetrators of IPV (Tjaden & Thoennes, 2000). Furthermore, terms such as domestic violence or family violence are often broadly used to refer to events occurring between family members (e.g., child and parent) or heterosexual couples who were legally married. Research has now clearly demonstrated incidents of IPV within relationships of co-habiting and divorced couples as well as within those in same sex relationships (Johnson & Ferraro, 2000). IPV as a term is more specific to violence occurring between intimate partners. The current review focuses on IPV in adults over the age of 18.

Adding an additional layer of complexity, early research on IPV focused on physical violence perpetration towards a partner at the expense of alternative forms of IPV (Golding, 1999). In turn, research tended to focus only on the physical consequences of IPV victimisation (Avant, Swopes, Davis, & Elhai, 2011). Developments in this field, both theoretical and empirical, have since led to a broadening of definitions which now recognise that IPV is multi-factorial. Current definitions include physical, sexual, and psychological acts of violence and coercive behaviour (Home Office, 2013). That being said, the UK definition is still based on the term domestic violence and does not distinguish clearly the differences between intimate partners or family members, rather the definition focuses on the act and the location. Alternatively, the US definition of IPV specifically represents violence among intimate partners (Centers for Disease Control and Prevention [CDC], 2014). Research following the US definition also considers stalking behaviour as a form of IPV (Tjaden & Thoennes, 2000). Such global disparities hinder researchers and practitioners in their attempt to understand the nature of IPV.

Walby (2009) suggested that the overall costs of IPV to society within the United Kingdom are estimated to be in excess of 10 billion pounds. These costs are accumulated through three key areas, (1) services, such as health care, social services, and the criminal justice system; (2) economic loss, for example, due to time off work caused by injury; and (3) human and emotional costs, that is, what individuals pay to avoid such injury (Walby, 2009). Earlier reports indicated that the annual cost of treating mental disorders as a result of IPV within the United Kingdom is £176,000,000 (Walby, Britain, & Britain, 2004). In a large scale US report provided by the Center of Disease Control, IPV costs were estimated to exceed $8.3 billion (Centers for Disease Control and Prevention [CDC], 2003). Of note, the health care costs for victims of IPV can continue for many years after the abuse has ended (Rivara et al., 2007).

In addition to the economic costs, exposure to IPV is known to be psychologically damaging (Lipsky et al., 2005a). Herman (1992) argued that violence perpetrated by an intimate partner may cause more psychological harm than violence perpetrated by a stranger. For example, when comparing IPV experience to alternative trauma experiences, IPV victims showed greater symptoms of posttraumatic stress disorder (PTSD) (Sharhabani-Arzy, Amir, & Swisa, 2005). It is proposed that IPV has a greater potential to affect one's mental health given that the violence is perpetrated by an individual whom the victim trusts (Herman, 1992). Knowing a perpetrator may be further damaging as a victim cannot justify the occurrence as a random anonymous attack rather a purposeful intent to hurt. The former provides a better vein in which to process and cope with the occurrence, with the latter further impinging upon mental health.

In a meta-analytic report provided by Golding (1999), it was suggested that mental health issues such as suicidal ideation, substance abuse, PTSD, and depression occur three to five times more frequently in survivors of IPV than in women who never experienced IPV (Golding, 1999). Such findings have been supported by many researchers who have similarly demonstrated that IPV has an adverse effect on mental health (Coker et al., 2002). Although Golding (1999) produced a comprehensive meta-analytic review, a clear focus was paid towards physical IPV at the expense of other types of IPV. This is surprising as although other acts such as psychological violence or emotional abuse within an intimate relationship may be more subtle and difficult-to-measure, they can still have far-reaching effects (Arias & Pape, 1999). One example is provided by Dutton, Goodman, and Bennett (1999) who reported that psychological violence is a strong predictor of PTSD. Dutton et al. (1999) examined court-involved IPV victim's traumatic responses to physical, sexual, and psychological violence. These different types of IPV were examined as predictors of PTSD. Univariate analysis indicated that all forms of violence were predictive of PTSD symptoms. However, a multivariate model indicated that psychological violence explained more variance in PTSD scores compared to physical violence. Cumulatively, psychological violence and level of injury increased the prediction of PTSD and acute stress symptoms. Psychological violence and level of injury explained 23% of the variance in PTSD and 37% of the variance in acute stress. Studies such as this indicate that psychological violence may be just as detrimental to mental health as other forms of IPV. This may be attributable to acts of psychological violence eliciting and maintaining internal feelings of fear, loss of control, and susceptibility to danger (Coker, Smith, Bethea, King, & McKeown, 2000).

Previous findings demonstrate that victims are often exposed to different forms of violence within a relationship, known as polyvictimisation (Sabina & Straus, 2008). For example, White and Smith in their longitudinal study (2004) found that women who had been victims of sexual violence in a relationship were also victims of physical violence. The co-occurrence of multiple types of violence confers greater risk to victims with regard to a greater number of adverse outcomes (Sabina & Straus, 2008). This is particularly salient in relation to mental health outcomes associated with IPV victimisation. Indeed, Armour and Sleath (in press) report that polyvictimisation of IPV across the life-course is associated with a greater degree of psychiatric morbidity.

IPV victimisation is further associated with the co-morbidity of mental disorders. For example, Stein and Kennedy (2001) found in their cross-sectional study of female IPV physical and/or sexual violence victims, that individuals reporting with PTSD symptoms also reported co-morbid major depressive symptoms (Stein & Kennedy, 2001). This is unsurprising given the similarities between a proportion of PTSD and depressive symptoms within the current diagnostic criteria, for example, sleeping difficulties. Indeed, research has questioned whether a number of symptoms across these two disorders may partly account for their co-morbidity (cf. Armour, McBride, Shevlin, & Adamson, 2010; Elklit, Armour, & Shevlin, 2010).

IPV and mental health symptoms are also known to relate to a number of indirect risk factors. Reports from a large scale World Health Organization study found that IPV victims with depressive symptoms were more likely to report drug and alcohol abuse as well as suicidal ideation (García-Moreno et al., 2005). Such behaviours may be the result of victims unknowingly trying to manage the adverse pain and emotion associated with IPV (García-Moreno et al., 2005). Within the same report, it was noted that severity and extent of violence within a relationship contributes towards the mental health outcomes of IPV victims. Victims who experience more than one form of violence and who are re-victimised are at increased risk of mental disorders and co-morbidity of disorders (García-Moreno et al., 2005).

Just as types of IPV may affect victim's mental health in different ways, gender may also play a part in mental health outcomes. During 2010/11, the British Crime Survey estimated that 30% of women (4.8 million) and 17% of men (2.8 million) had experienced IPV between the ages of 16 and 59. A recent report provided by the Office for National Statistics (ONS, 2013) showed that 7.3% of women and 5% of men reported having experienced IPV in the past year (2011/12). Although a considerable amount of research to date has highlighted that IPV is not restricted to one gender, it is also widely noted that females are at increased risk of repeat victimisation and are more likely to suffer from the adverse outcomes associated with the various types of IPV (Coleman, Jansson, Kaiza, and Reed, 2007). That being said, research has also highlighted that male victims may endure the more subtle types of violence such as psychological violence (Dutton & Nicholls, 2005). As noted earlier, this may be just as adverse as other forms of violence. Furthermore, males are less likely to report or respond to questions related to their IPV victimisation (Brown, 2004). Women, due to differences in size and strength, are also more at risk of receiving physical injury compared to males (Kaur & Garg, 2008). For such reasons, little consideration has been given to gender outcomes in relation to IPV and mental health with a large focus on female victims and mental health outcomes (Próspero, 2007).

It is the aim of this review to add to previous knowledge provided by Golding (1999) by considering the mental health outcomes associated with IPV victimisation in relation to all forms of IPV. Where possible, the current review also aims to explore gender similarities and differences.

## Methodology

The initial framework for this review was designed to broadly search and find relevant literature aimed at addressing the question, “how does intimate partner violence affect the mental health of victims experiencing any form of IPV.” Medical Subject Headings (MeSH) and text words were used to search 11 Biomedical and Social Science electronic databases from 2004 until the current date (February, 2014). Search protocols and key word terms for IPV and mental health were adopted from previous literature reviews (see, Trevillion, Oram, Feder, & Howard, 2012). A hand search of the journal *Trauma, Violence, & Abuse* as well as Internet search engines (Google, Google Scholar) were also explored for research related to IPV and mental health outcomes. Searches were limited to include articles published in the past 10 years and written in English. Searched databases are as follows:

- Biomedical database: CINAILL, Cochrane, EMBASE, MEDLINE (Ovide), PsycINFO, Science Direct, Web of Science (including SCI-EXPANDED, SSCI, A&HCI, CPCI-S, CPCI-SSH).
- Social science database: Applied Social Sciences Index and Abstracts (ASSIA), International Bibliography of the Social Sciences (IBSS) AND Sociological Abstracts
- Thesis and Dissertations: DART Europe E Thesis Portal
- Manual search of *Trauma, Violence, & Abuse*
- Internet Search engines: Google & Google scholar

An example of a search strategy used for EMBASE includes search terms “intimate partner violence” (MeSH Term) or “partner violence” (All fields), “domestic violence” (All fields), “battered woman” (All fields), 1 OR 2 OR 3. “Mental health” (All fields), “mental disorder” (MeSH Term), “mentally ill persons” (MeSh Term), 4 OR 5 OR 6. “Anxiety disorder” (All fields), “anxiety” (All fields), “depression” (MeSH Term), OR “depression” (All Fields) OR “major depression” (All fields), “sleep disorder” (All Fields), “posttraumatic stress disorder” (All Fields), “eating disorder” (All fields), 7 OR 8 OR 9 OR 10 OR 11 OR 12 OR 13 AND 14 AND 15, limited to (human and English language and adult >18 to 64 years< and past 10 years). Key word searches were also used within databases where advanced search options were not available.

### Study eligibility

*Inclusion criteria:* studies were included if they incorporated adult individuals both male and female, ≥18 years, who had experienced IPV (life time and past year) and whose mental health had been measured using a valid standardised measurement tool. Study samples “males only,” “females only,” or “a mix of both” were included in the review. Studies which presented results of research based on experimental design (e.g., randomised control trials, non-randomised control trials), cross-sectional, or intervention-based studies, were also included. This will mean that not all included studies will use a comparative group such as an IPV group and non-IPV group.

*Exclusion criteria:* studies were excluded if their samples included individuals under 18 years or included participants with special disabilities or certain complicated diseases, for example, HIV. Also, studies focusing on IPV during pregnancy were excluded, given that “such abuse is thought to differ in important ways from battering at other times” (Golding, 1999, p. 103). Furthermore, studies were also excluded if they were published in excess of 10 previous years, included individuals with pre-existing mental disorders, and were published in a foreign (non-English) language.

**Fig. 1:**
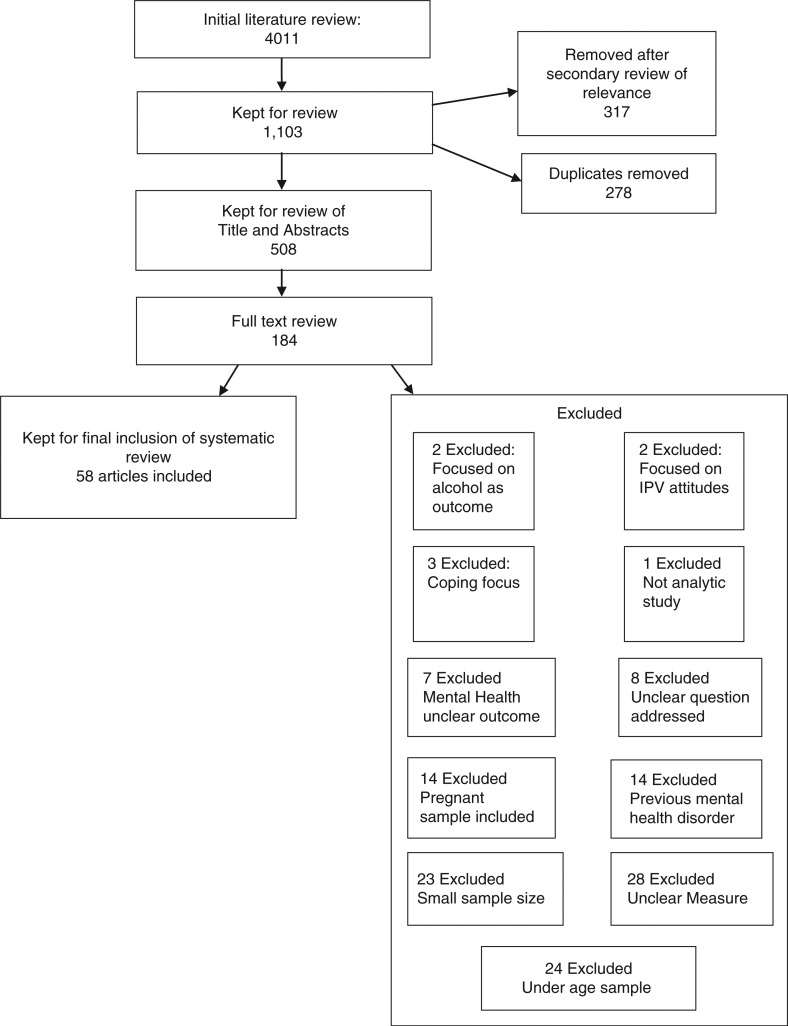
Flowchart of study selection.

Two reviewers agreed on the search terms to be included. Reviewer one conducted the first literature search. Using the search terms described above, the initial literature search produced 4,011 articles. Article titles were screened for eligibility with the most relevant articles retained for abstract review. After an initial review of relevance, 1,103 articles were retained for further review of their titles and abstracts. Reviewer two also screened articles independently of reviewer one. A further 317 articles were removed based on second observations deeming them not relevant. Article references and abstracts were transferred to a web-based reference management software system (RefWorks) and duplicates were removed. When article abstracts provided insufficient information, the full text was obtained, if possible, for further consideration. Five hundred and eight articles were retained for abstract review. Based on eligibility criteria, 184 full text articles were assessed for quality appraisal using criteria adopted from the Critical Appraisal Skills Programme (CASP, 2012). CASP tools are generally employed in order to ensure the quality of papers to be included within a review. Key questions for consideration are laid out for the researcher to follow during review of papers. Two reviewers assessed the full text articles using criteria adopted from the CASP tool. The reviewers then met and an inter-rater agreement of inclusion of studies was made based on quality and eligibility criteria. After a final review of the full text articles, 58 studies were included for the systematic literature review (Cavanaugh et al., 2012 & Lipsky, Field, Caetano and Larkin, 2005b). (See Fig. 1.) for overview of study selection.

Two reviewers extracted data, including information relating to the authors, publication date, sample population, size, gender division, study design, IPV type, IPV measure, mental health outcome addressed, mental health measure, and key results. These study features were addressed as previous research has noted that they may potentially account for differences across study results (Golding, 1999). Data from the 58 studies were not sufficiently homogenous to allow statistical pooling. Many of the studies employed a variation of statistical methods which do not lend themselves to comparison across studies. Alternatively a narrative synthesis was conducted in order to identify relevant information to address the research questions (see O'Reilly, Beale, & Gillies, 2010). Data of the main findings of individual studies can be found in Supplementary Table 1.

## Results

### Studies’ characteristics

A total of 58 studies were included in the final review (see Supplementary Table 1 for the extracted data noted above. Full references for included studies can also be found in the reference list.). A large majority of these studies were cross-sectional in design (83%) and came from the United States (74%) (see Supplementary Table 1; studies: 1, 2, 3, 4, 5, 6, 7, 10, 11, 12, 14, 15, 17, 18, 20, 21, 22, 23, 25, 26, 27, 28, 29, 31, 34, 35, 36, 39, 41, 42, 43, 44, 45, 46, 47, 48, 50, 51, 52, 54, 56, 57, 58). These were followed by several studies from a number of geographical locations, including Asia, New Zealand, and Europe (studies: 8, 9, 13, 16, 19, 24, 30, 32, 33, 37, 38, 40, 49, 53, 55). Most studies focused on female samples (62%) and female victims (26%) with a small group considering both males and females (31%). Four studies (21, 23, 44, 45) focused on males alone (7%). A total of 141,319 participants were included across all study samples. The majority of these studies assessed mental health outcomes often cited in the literature. The key mental health outcomes included depression, PTSD, anxiety, and general psychological distress.

### Depression

The highest number of papers in this review reported on the relationship between IPV and depression (studies: 1, 6, 7, 8, 9, 10, 12, 14, 15, 16, 17, 19, 20, 22, 23,25, 26, 27, 29, 30, 31, 33, 35, 36, 37, 38, 39,40, 41, 43,44, 45, 47, 51, 52, 53, 55, 57, 58). Depression was noted as a significant outcome of experiencing IPV across a variety of samples. Studies which compared victims with non-victims of IPV indicated that depression was much more prevalent among IPV victims (2, 6, 7, 14,17, 19, 22, 23,25, 30, 33, 35, 37, 38, 40, 44, 45, 58) with the exception of one study (15). Twenty-six studies considered the relationship between the different types of violence (physical, psychological/emotional, sexual, and stalking), and the severity of violence exposure (repeated exposure or more than one type of violence), with the development of depression. Psychological, physical, and sexual violence were found to have an effect on depression as a result of IPV (studies: 6, 8, 9, 10, 14,17, 19, 20, 22, 23,25, 26, 29, 31, 36, 37, 38, 40, 42, 43, 47, 51, 52, 55). That being said, a large number of studies found that psychological violence alone had an effect on depressive outcomes (9, 25, 26, 31, 38, 43, 51, 55), with three studies reporting that psychological violence had an independent effect on depressive outcomes after controlling for other types of violence (26, 38, 55). Studies that accounted for polyvictimisation found that repeated exposure to different forms of violence within a relationship increased the risk of depression across various samples (6, 7, 10, 40, 42, 46). A total of 18 studies also assessed the co-morbidity of depression with other disorders. Results indicated that depression co-occurred on a number of occasions with PTSD (studies: 15, 27, 29, 31, 35, 37, 38, 47, 53). Relationships between the co-occurrence of depression and anxiety, depression and suicide, depression and sleep disturbance, and depression and drug abuse in relation to IPV victimisation were also noted (studies: 2, 8, 9, 12, 25, 30, 39, 43, 56).

### Posttraumatic stress disorder

A total of 33 studies indicated that of individuals assessed for experiencing some form of IPV, PTSD was considered a significant outcome (1, 3, 4, 5, 7, 10, 14, 15, 18, 21, 22, 26, 27, 28, 28, 29, 31, 33, 34, 35, 36, 37, 38, 39, 45, 46, 47, 48, 50, 53, 54, 56, 57). Studies which compared victims of IPV with non-victims (7, 14, 15, 22, 33, 34, 35, 36, 37, 37, 45, 48, 57) found that IPV victims were much more likely to display symptoms of PTSD compared to trauma exposed non-IPV victims. A number of studies also considered the different types of violence and the severity of violent exposure within a relationship that may impact on the risk of PTSD development. A large number of studies reported that experiencing physical and psychological violence within a relationship significantly contributed to the probability of reporting PTSD symptoms compared to other forms of violence (5, 7, 14, 27, 37, 38, 46). Furthermore, those studies that controlled for particular types of violence and the influence of each type of violence on PTSD found that psychological violence alone was a key predictor of PTSD (31, 34, 38). Studies that accounted for polyvictimisation found that repeated exposure to different forms of violence within a relationship increased the risk of PTSD across various samples (7, 10, 22, 28, 29, 31, 38). Twelve studies (15, 27, 29, 31, 35, 37, 38, 39, 47, 53), reported that PTSD is significantly associated with depressive symptoms and, in two studies (56, 57), a predictor of sleep disturbance. Fewer studies noted that PTSD as a result of IPV can also be linked to drug problems (study 50) and suicidal ideation (study 28). The severity of IPV was also associated with the development of PTSD and co-morbidity of PTSD with depression (studies: 35, 47).

### Anxiety

A total of 15 studies (1, 9, 12, 16, 20, 30, 33, 36, 37, 38, 41, 42, 43, 51) found that individuals experiencing IPV reported symptoms of anxiety, with victims reporting severe symptoms than non-victims (studies: 30, 33, 36, 37, 38). Psychological, physical, and sexual violence were found to be associated with anxiety, with a higher number of studies indicating that psychological violence exposure was associated with anxiety (26, 30, 37, 43, 51). Severity of IPV exposure was also found to increase symptoms (studies: 12, 37, 41). A small number of studies found that anxiety and depression tended to co-occur as a result of IPV (9, 30). Anxiety was also found to be associated with sleep disturbance in IPV victims (studies: 12, 43).

### Psychological distress

Six studies measured IPV and its association with psychological distress (2, 11, 13, 32, 49, 51). The term psychological distress has been used to describe an overall measure of psychological symptoms such as depression, anxiety, somatisation, interpersonal sensitivity, and hostility. Psychological distress was reported as the global measure of symptoms as reported by victims of IPV. Studies using a measure of psychological distress found that victims of IPV were more likely to report higher psychological distress compared to non-victims (2, 11). Physical and psychological violence (studies: 11, 13, 49, 51) and severity of violence (13) were noted as contributing towards feelings of psychological distress among victims.

### Somatisation

Three studies also considered the effect of IPV victimisation and somatic symptoms (20, 41, 42). Somatisation is the physical manifestation of psychological distress. Psychological, physical, and sexual violence were found to be related to somatic symptoms amongst victims (studies: 20, 42). Severity of IPV also increased the probability of displaying somatic symptoms (42).

### Male & female comparisons

Sixteen studies included both males and females in their sample (1, 3, 8, 11, 12, 16, 19, 26, 41, 42, 43, 46, 49, 51, 52, 53). Of these, seven studies (3, 8, 16, 41, 42, 43, 46) stated that both males and females reported similar rates of IPV victimisation with the exception of (study 11) who found higher victimisation reports in females. A number of studies also noted that female IPV victims were more likely to experience anxiety and depression in comparison to males (1, 12, 16, 43, 49). Four studies also compared males and females on the effects of types of violence and mental health outcomes. Four studies (1, 8, 19, 53) found that physical violence affected both men and women in relation to symptoms of anxiety, depression, PTSD, and suicidal ideation. Three studies (3, 8, 46) noted that women exposed to physical violence were more likely to report PTSD symptoms and depression compared to males. Four studies (26, 42, 46, 51) found that as a result of psychological violence, men and women reported symptoms of anxiety and depression. Two studies (46, 51) found that men displayed higher anxiety scores than females. A number of studies found that IPV victimisation and suicidal ideation was similar among males and females (1, 8). Furthermore, experiencing anxiety and depression as a result of victimisation was associated with reports of sleep disturbance in both males and females (studies: 12, 43). Severity of IPV also contributed towards increased mental health symptoms in both males and females (study 52).

### Childhood influence on IPV victimisation and mental health

Although not a key focus of this review, it is worth noting that several studies also measured the prevalence of childhood abuse and its relationship with IPV victimisation and mental health outcomes (1, 6, 7, 16, 17, 28, 37, 44, 48, 57). It was noted that individuals reporting IPV victimisation were also more likely to report experiencing childhood abuse or observing IPV as children compared to non-victims. Also, those who had experienced or observed abuse as children were more likely to report later IPV victimisation and mental health outcomes such as depression, PTSD, and sleep disturbance.

## Discussion

This review included papers assessing different forms of IPV in an attempt to clarify if the type of IPV experienced was an important factor in mental health outcomes. Although physical violence and a combination of physical and psychological violence were related to adverse mental health outcomes, the relationship between psychological violence and mental health are noteworthy. Indeed, a lack of clear validated measures assessing the impact of psychological violence has meant that researchers have not clearly focused on this type of violence (Lawrence, Yoon, Langer, & Ro, 2009). Researchers may also find it difficult to separate psychological violence from other forms of violence as in many cases psychological violence occurs in the presence of other forms of violence (Sabina & Straus, 2008). Moreover, psychological violence has previously been viewed as having less impact than other forms of violence (Lawrence et al., 2009).

Psychological violence (also called psychological/emotional abuse, verbal abuse or aggression, nonphysical abuse or aggression) can be characterised by verbal and non-verbal acts which are used to threaten, terrorise, intimidate, belittle, control, and diminish an individual in order to limit and destroy self-esteem and well-being (Saltzman, Fanslow, McMahon, & Shelley, 2002). Research suggests that this type of violence has a greater impact on victims’ mental health as it maintains an abusive relationship (Sackett & Saunders, 1999). Of note, internalising feelings such as self-doubt and fear may cause individuals to stay within an abusive relationship. Those subject to psychological violence have been compared to prisoners of war in that victims are indoctrinated into a violent culture. As a result, individuals report a loss of identity and control which can lead to feelings of hopelessness and an inability to leave the abusive relationship (Sackett & Saunders, 1999). Psychological violence, similar to IPV, has a number of different facets; research has noted that the dominance/intimidation and belittling components of psychological violence are significant unique predictors of PTSD and psychological distress (Norwood & Murphy, 2012). In addition, Wilson et al. (2011) noted that “… fear is a powerful predictor of PTSD” (p. 24). This is pertinent in the relationship between IPV as it relates to PTSD, given that psychological violence can create an environment whereby a victim waits in fearful anticipation for the next violent act to occur.

Psychological violence can also result in individuals being restricted from social support, which further diminishes identity and control (Escriba-Aguir et al., 2010). Again, this is pertinent in the link between psychological IPV and subsequent PTSD given that social support in the aftermath of traumatic experience is a known protective factor in the development of PTSD symptoms (Karstoft, Armour, Elklit, & Solomon, 2013). Early qualitative research such as Walker (1984) and Follingstad et al. (1990) found that IPV victims reported psychological violence as more harmful than other types of violence (Lawrence et al., 2009). Psychological violence as a predictor of mental health still receives less attention than other forms of violence within research (Pico-Alfonso et al., 2006). Based on the current review, it can be noted that psychological violence can no longer be considered a minor type of violence but rather a possible key predictor of certain mental health outcomes.

A review of the literature also highlighted that IPV victimisation can result in multiple psychiatric morbidities. Indeed, across studies, IPV was significantly related to depression, PTSD and anxiety. The literature suggests that depression tends to co-occur most often with PTSD. The inter-relationships between these two psychiatric morbidities have been confirmed in studies by Contractor et al. (2014), Biehn et al. (2013), and Armour et al. (2012).

Depression, PTSD, anxiety, and co-morbid disorders were also found to co-occur with indirect outcomes such as drug abuse, suicidal ideation, and sleep disturbance. The exploitation of substances by victims is said to be a means by which individuals attempt to self-medicate and cope with an inevitable situation (Carbone Lopez, Kruttschnitt, & Macmillan, 2006). Leiner, Compton, Houry, and Kaslow (2008) suggested that suicidal ideation is the result of a particular mental health pathway. She suggested that the interaction between PTSD and depression can increase the likelihood of suicidal ideation. Moreover, symptoms of co-morbid disorders were also found to increase with the severity of IPV (O'Campo et al., 2006; Sabri et al., 2013); which in turn further affects a victim's ability to manage, function, and cope in a hostile environment.

Very little previous research has considered the association between IPV, mental health, and sleep disturbance. In this review, only a small number of studies reported on the associated mental health symptoms and sleep disturbance in IPV victims (39, 43, 56, 57, 12). In particular, co-morbid anxiety and depression was found to impact on sleep quality in IPV victims (El-Sheikh, Kelly, & Rauer, 2013; Rauer, Kelly, Buckhalt, & El-Sheikh 2010). This is unsurprising given that sleep “… is an inherently vulnerable state that is facilitated by feelings of safety in one's environment” (Rauer, et al., 2010, p. 1). Thus, as feelings of fear are associated with IPV victimisation, living in fearful conditions will interfere with an IPV's victim's quality of sleep. Sleep quality has been noted as having an important restorative function in both physical and psychological recovery (Woods, Kozachik, & Hall, 2010). Such functions are particularly important for IPV victims because of the repeated exposure to IPV. Individuals who are unable to achieve quality sleep may be at greater risk of psychiatric morbidity and thus will have a reduced ability to cope with repeated IPV exposures.

The literature suggested that both males and females are at risk of the mental health outcomes associated with IPV experience. That being said, some of the literature has suggested that the mental health of IPV victims may be affected in different ways depending on gender. For example, depending on the type of violence exposure, females subjected to physical violence were more likely to display higher PTSD and depressive symptoms compared to males (Chan, Straus, Brownridge, Tiwari, & Leung, 2008; Sabina & Straus, 2008). Males were more likely to display anxiety symptoms in relation to psychological violence compared to females (Sabina & Straus, 2008; Taft et al., 2006). As noted earlier, if the majority of studies are assessing physical violence exposure, then adverse outcomes, particularly those related to mental health, may not be apparent in relation to males. Moreover, it is suggested that females are more likely to perpetrate psychological violence against males rather than physical violence (Straus, Hamby, Boney-McCoy, & Sugarman, 1996). If this type of violence is not adequately measured throughout the research, less will be known about the impact of IPV on male victim's mental health.

A number of studies also considered the pre-exposure of victims to childhood observations and experiences of violence. Reflective of a large vein of existing research (e.g., Akbar Rahmatian, 2009), those who had experienced or observed violence as children were more likely to report IPV victimisation as adults. These individuals were also more likely to report mental health outcomes compared with those who had never experienced lifetime exposure (both childhood & adult victimisation). Avant et al. (2011) suggested that increased mental health outcomes in these individuals can be explained through cumulative stress theory, which proposes an increasing build-up of stress with each additional stressful event/trauma (Rutter, 2012). Also, if individuals are continually exposed to violence through those with whom they are interpersonally connected, greater detriment to well-being may be inevitable. For example, La Flair, Bradshaw, and Campbell (2012) found that female IPV victims who also had a history of childhood trauma displayed steady depression scores over time, even after the relationship had ended. Thus, the accumulation of violent exposures may have lasting effects beyond the present experience.

### Limitations

The current review extends beyond previous reviews in that all types of violence and mental health outcomes were considered where possible. That being said, only a small number of studies included in this review considered lifetime exposure to violence including that which occurred in childhood. The remainder of studies did not take this important factor into consideration and thus did not control for its potential influence on mental health reports. The studies included in this review also used various measures of both IPV and mental health outcomes making definitive conclusions difficult. Much of the literature relied on self-report measures and did not consider the context in which the violence occurred. The review was limited to literature published in the past 10 years and only included adults over the age of 18. This may have resulted in a loss of relevant information. Also, the current study did not address cross cultural comparisons; this would be a useful endeavour in future research.

## Conclusions

The current paper has considered collectively the importance of a number of high impact factors associated with the field. The factors systematically explored through this piece include considerations for types of violence, gender issues, intergenerational transmission of violence, and mental health outcomes as they relate to IPV experience. We have attempted to demonstrate the importance of considering and measuring accurately the various forms of violence within IPV research. More attention should be directed towards the effects of psychological violence and how it influences mental health. The review has also demonstrated that gender differences in IPV should be given more attention in terms of mental health outcomes. “Little is known about the developmental course of psychological aggression or its impact on individual's well-being in non-battering populations” (Lawrence et al., 2009, p. 23). Defining pathways leading from various IPV experiences and how they relate to psychiatric morbidities should be of great interest to both researchers and practitioners, particularly in relation to future intervention developments. A review of the literature has also helped highlight the importance of considering the indirect impacts of IPV such as sleep disturbance; this area is particularly neglected in the field. Finally, literature obtained through the process of this review came from a diverse range of cultures and samples. Researchers and policy makers may both have a vested interest in the development of an agreed global definition of the term IPV. An agreed definition would allow for greater comparisons and understanding of the true nature of IPV.
